# Evaluation of patients with behavioral and cognitive complaints: misdiagnosis in frontotemporal dementia and Alzheimer's disease

**DOI:** 10.1590/S1980-57642013DN70100010

**Published:** 2013

**Authors:** Bárbara Costa Beber, Márcia L.F. Chaves

**Affiliations:** 1 Dementia Clinic, Neurology Service, Hospital de Clínicas de Porto Alegre (HCPA), Porto Alegre RS, Brazil. Postgraduate Program in Medical Sciences, Faculty of Medicine, Universidade Federal do Rio Grande do Sul (UFRGS), Porto Alegre RS, Brazil. CAPES PhD scholarship.; 2 Dementia Clinic, Neurology Service, HCPA. Postgraduate Program in Medical Sciences, Faculty of Medicine, UFRGS. Department of Internal Medicine, Faculty of Medicine, UFRGS.

**Keywords:** frontotemporal dementia, Alzheimer's disease, diagnosis

## Abstract

**BACKGROUND:**

Frontotemporal dementia (FTD) is a heterogeneous clinicopathological syndrome whose early diagnosis is critical for developing management strategies.

**OBJECTIVE:**

To analyze the variables associated with misdiagnosis in a group of patients with FTD, Alzheimer's disease (AD), and without neurodegenerative disorders (WND), all of whom were evaluated for behavioral and cognitive complaints.

**METHODS:**

A case-control study with FTD (n=10), probable AD (n=10) and WND (n=10) patients was carried out. The studied variables were disease duration, reason for referral, former diagnosis, behavioral and cognitive symptoms at evaluation, MMSE at the specialist evaluation, and follow-up outcome. The data were analyzed by ANOVA with Bonferroni *post-hoc* and by Pearson's Chi-Square tests.

**RESULTS:**

FTD patients and WND patients showed longer disease duration than AD patients; the main reasons for referral in the FTD group were behavioral, memory and memory plus language problems while all AD and 90% of the WND group were referred for memory. The FTD group had the highest rate of misdiagnosis and worst outcomes after the 12-month follow-up. The majority of AD and WND patients had memory symptoms, while FTD patients presented language (30%), memory and/or language (40%) problems on the evaluation.

**CONCLUSION:**

Difficulty in recognizing the main features of FTD and psychiatric disorders with memory impairment was observed. Clinicians tended to generalize memory complaints toward a single diagnosis, identifying almost all these patients as AD or leaving them undiagnosed.

## Abstract (pt)

**INTRODUÇÃO:**

Demência frontotemporal (DFT) é uma síndrome clinicopatológica heterogênea e seu diagnóstico precoce é essencial para o desenvolvimento de estratégias de manejo.

**OBJETIVO:**

Analisar as variáveis associadas ao erro dignóstico em pacientes com DFT, doença de Alzheimer (DA) e sem transtornos neurodegenerativos (STN), avaliados por queixas cognitivas e comportamentais.

**MÉTODOS:**

Estudo de caso-controle foi realizado com pacientes com DFT (n=10), provável DA (n=10), e STN (n=10). As variáveis estudadas foram duração da doença, motivo do encaminhamento, diagnóstico prévio, sintomas cognitivos e comportamentais na avaliação especializada, MEEM na avaliação, e desfecho. As análises foram feitas por ANOVA com Bonferroni *post-hoc* e Qui-Quadrado de Pearson.

**RESULTADOS:**

Pacientes com DFT e STN mostraram maior tempo de duração da doença; os principais motivos de encaminhamento no grupo DFT foram problemas comportamentais, memória e memória mais linguagem, enquanto em todos pacientes com DA e 90% do grupo STN foi memória. O grupo DFT teve maiores taxas de erro diagnóstico e piores desfechos no seguimento de 12 meses. A maioria dos pacientes com DA e STN teve sintomas de memória, enquanto pacientes com DFT apresentaram sintomas de linguagem (30%), memória e/ou linguagem (40%) na avaliação.

**CONCLUSÃO:**

Dificuldade em reconhecer as principais características da DFT e de transtornos psiquiátricos com prejuízo de memória foi observada. Os clínicos tenderam a generalizar queixas de memória em direção a um único diagnóstico, identificando quase todos estes pacientes como tendo DA ou deixando-os sem diagnóstico.

## INTRODUCTION

Frontotemporal dementia (FTD) is a heterogeneous clinicopathological syndrome with progressive degeneration of the frontal lobes, anterior temporal lobes, or both. FTD patients make up about 10% of all patients with dementing diseases. Because FTD is usually a presenile onset disorder, it accounts for approximately 20% of neurodegenerative dementias among dementia patients with age at onset of less than 65 years.^1-3^ In Brazil, FTD accounts for about 5% of presenile dementia cases.^4^

The characterization of the clinical types of FTD have evolved from the first consensus on diagnostic criteria (Lund and Manchester research criteria, 1994)^5^ with three FTD symptom constellations: [1] behavioral symptoms, [2] affective symptoms, and [3] speech disorder, to the Neary and colleagues (1998)^6^ diagnostic criteria encompassing three distinct clinical variants that can be distinguished based on the early and predominant symptoms: a behavioral-variant (bvFTLD) and two language variants (semantic dementia and progressive nonfluent aphasia), and finally to the two 2011 consensus on diagnostic criteria^7,8^ establishing four different subdivisions: [1] a frontal or behavioral variant (bvFTLD); [2] SD or Semantic variant Progressive Primary Aphasia (PPA-semantic); [3] PNFA or Nonfluent/agrammatic variant PPA (PPA-agrammatic); and [4] logopenic progressive aphasia or Logopenic variant PPA (PPA-logopenic).

FTD is often misdiagnosed and, among the other neurodegenerative disorders, is commonly mistaken for Alzheimer's disease (AD).^9,10^ The main difference between the two types of dementia is the presence of changes in personality, motivation, social interaction and organizational abilities, in the presence of well-preserved memory and visuospatial abilities in FTD. On the other hand, AD is characterized by a progressive amnestic disorder with episodic and semantic memory deficit, followed by breakdown in other attentional, perceptual and visuospatial abilities.^11^ Many of FTD's initial symptoms, albeit behavior or language related, are compatible with a range of neurologic disorders and because FTD often affects people in midlife it is also frequently mistaken for primary psychiatric disorders such as depression or psychosis.^10,12,13^

In bvFTD, neuropsychiatric changes are the most prominent symptoms and usually precede or overshadow cognitive disabilities, whereby changes in personality and behavior observed by the family often go unnoticed by the majority of patients. Suspicion of FTD arises when there is a gradual personality change and frontotemporal abnormalities on neuroimaging, particularly frontotemporal hypometabolism.^1,8,12^

Early diagnosis of FTD is critical for developing management strategies and interventions, but clinicians or general practitioners continue to have difficulty diagnosing early FTD. Without a definitive clinical test, the early diagnosis of FTD can be challenging. Consequently, patients with FTD can go from physician to physician delaying diagnosis and jeopardizing therapy. Despite this diagnostic confusion, there is scant data on the accuracy of a clinical evaluation for FTD.^13^

The objective of this study was to analyze variables associated with misdiagnosis in FTD and AD patients, and in a group without neurodegenerative disorders (WND). All patients were evaluated for behavioral and/or cognitive complaints.

## METHODS

A case-control study including ten patients with FTD, 10 patients with probable AD and 10 patients WND was carried out. All patients were selected during the same period and samples were balanced for criteria of entry into the study for the period (August/2009 to August/2011) limiting inclusion to "typical" cases in each category after expert evaluation. "Typical" cases were defined as those patients who were evaluated for the first time at the specialized outpatient clinic and presented sufficient clinical and laboratory data to fulfill AD diagnostic criteria or to exclude the presence of neurodegenerative disorders. During the period of the study, 100 new (first) evaluations were carried out, and 10 AD and 10 WND were found among these subjects. The patients were selected from the Dementia Clinic of the Hospital de Clínicas de Porto Alegre (HCPA).

The 1998 consensus diagnostic criteria for FTD were applied,^6^ and the National Institute of Neurologic and Communicative Diseases and Vascular Cerebral Accident and Alzheimer Disease Related Association (NINCDS-ADRDA) criteria were used for probable AD.^14^

The studied variables were disease duration, reason for referral, former diagnosis, behavioral and cognitive symptoms at the specialist evaluation, MMSE score at the specialist evaluation, and follow-up outcome. Severity of disease (CDR scale) and use of cholinesterase inhibitors and/or memantine were also recorded.

This study was approved by the Human Research Ethics Committee and signed consent was obtained from all patients or a proxy.

The continuous variables were expressed as mean and standard deviation and analyzed by the one-way ANOVA with the Bonferroni *post hoc* test. The categorical variables were expressed in absolute and relative frequency and data were analyzed by Pearson's Chi-Square test. All analyses were considered statistically significant at a p-value <0.05.

## RESULTS

Demographic and clinical data of the samples are given in Table 1. Patients with AD were older than FTD patients and WND patients. AD and FTD patients had significantly lower MMSE scores. FTD patients and WND patients showed longer disease duration than AD patients.

**Table 1:** Demographic and clinical data of the groups studied

Variables	FTD (n=10)	AD (n=10)	WND (n=10)
Age	65.80+2.13^a^	78.30+2.13^a,b^	66.30+2.13 ^b^
Sex (male)	5 (55.6%)	3 (30%)	2 (20%)
Education	10.70+1.63	4.89+1.72	4.90+1.63
MMSE	11.19+1.35^c^	13.67+1.13^d^	26.31+0.94^c,d^
Disease duration	4.40+0.66^e^	1.80+0,66^e,f^	4.33+0,70^f^
Diagnosis	bvFTD 4 (40%) PPA 6 (60%)	AD 10 (100%)	Depression 7 (70%) No disorder 2 (20%) Undefined 1 (10%)

FTD: Frontotemporal Dementia; AD: Alzheimer's disease; WND: without neurodegenerative disorders; bvFTD: behavioral variant of Frontotemporal Dementia; PPA: primary progressive aphasia; MMSE: Mini-Mental State Examination;

a,b p=0.001 (Bonferroni *post hoc*);

c,d p<0.001(Bonferroni *post hoc*);

e p=0.03 (Bonferroni *post hoc*);

f p=0.042 (Bonferroni *post hoc*).

All AD patients and 90% of the WND patients were referred due to memory problems, while FTD patients were referred due to behavioral (30%), memory (30%) and memory plus language (30%) problems. Eighty percent of AD patients arrived for evaluation at the specialized clinic with no diagnosis. FTD patients arrived with the diagnosis of AD (30%), depression (20%), and mania (20%). WND patients were referred with suspected depression (30%), no diagnosis (30%), and AD (20%) (Table 2). No diagnosis (40%) and AD (23%) were the most frequent diagnoses based on results of the evaluation of the three groups.

**Table 2:** Reason for referral, former diagnosis and follow-up outcome of the groups studied.

	Variables	FTD (n=9)	AD (n=10)	WND (n=10)
Reason for referral*	Behavioral	3 (30%)	0 (0%)	1 (10%)
	Communication	1 (10%)	0 (0%)	0 (0%)
	Memory	3 (30%)	10 (100%)	9 (90%)
	Memory and communication	3 (30%)	0 (0%)	0 (0%)
Former diagnosis**	Without diagnosis	1 (10%)	8 (80%)	3 (30%)
	Depression	2 (20%)	0 (0%)	3 (30%)
	Mania	2 (20%)	0 (0%)	0 (0%)
	Alzheimer's disease	3 (30%)	2 (20%)	2 (20%)
	Psychotic disorders	0 (0%)	0 (0%)	1 (10%)
	Frontotemporal dementia	1 (10%)	0 (0%)	0 (0%)
	Indefinite	1 (10%)	0 (0%)	1 (10%)
Follow-up outcome***	Death	2 (20%)	0 (0%)	0 (0%)
	Institutionalization with worsening	2 (20%)	0 (0%)	0 (0%)
	Remained at clinic with worsening	4 (40%)	6 (60%)	0 (0%)
	Remained at clinic stable	2 (20%)	4 (40%)	0 (0%)
	Discharged	0 (0%)	0 (0%)	10 (100%)

FTD: Frontotemporal Dementia; AD: Alzheimer's disease; WND: without neurodegenerative disorders;

* p=0.017;

** p=0.099;

*** p=0.000 (Pearson's Chi-square).

After a 12-month period of follow-up at the clinic, 60% of AD patients were still followed and worsened. All WND patients were discharged from the Dementia Clinic and referred for specific management when necessary. Of the FTD patients, 40% were followed and worsened, 20% were institutionalized and worsened, and 20% died (Table 2).

Seventy percent of AD patients and 90% of the WND patients presented memory symptoms at the specialist evaluation, while FTD patients presented language (30%), memory (20%) and memory plus language (20%) symptoms. Of the 10 AD patients, 7 presented no behavioral symptoms. Half of the WND patients presented depressive or anxious symptoms. FTD patients presented no behavioral symptoms(40%), depressive or anxious symptoms (20%), loss of social adequacy (20%) and psychotic symptoms (20%) (Table 3).

**Table 3:** Cognitive and behavioral symptoms at specialist evaluation of the groups studied.

	Variables	FTD (n=9)	AD (n=10)	WND (n=10)
Cognitive symptoms at specialist evaluation*	Absent or not reported	1 (10%)	0 (0%)	1 (10%)
	Language	3 (30%)	0 (0%)	0 (0%)
	Memory	2 (20%)	7 (70%)	9 (90%)
	Critical judgment and abstraction	1 (10%)	0 (0%)	0 (0%)
	Memory and orientation	1 (10%)	3 (30%)	0 (0%)
	Language and memory	2 (20%)	0 (0%)	0 (0%)
Behavioral symptoms at specialist evaluation**	Absent or not reported	4 (40%)	7 (70%)	4 (40%)
	Depressive or anxious	2 (20%)	1 (10%)	5 (50%)
	Loss of critical judgment, social inadequacy (loss of insight and judgment)	2 (20%)	0 (0%)	0 (0%)
	Psychotic symptoms	2 (20%)	2 (20%)	0 (0%)
	Depressive and psychotic symptoms	0 (0%)	0 (0%)	1 (1%)

FTD: Frontotemporal Dementia; AD: Alzheimer's disease; WND: without neurodegenerative disorders;

* p=0.022;

** p=0,132 (Pearson's Chi-square).

According to the CDR scale, 29% of FTD patients were moderate, 57% severe, and 14% no dementia. In the AD group, 75% were mild and 25% moderate. None of the WND patients had dementia (CDR=0).

Of the 10 patients with FTD, five were using cholinesterase inhibitors and two, memantine. Only one of the AD patients had previously received a cholinesterase inhibitor. One WND patient was using memantine.

## DISCUSSION

The present study was carried out to evaluate the variables associated with misdiagnosis of FTD and AD in patients evaluated for behavioral and cognitive symptoms. Our main findings were a high rate of misdiagnosis prior to the specialist visit, longer duration of symptoms until specialized evaluation in both FTD and WND groups, and that AD was the main misdiagnosis in the FTD group.

The FTD group had the highest rate of misdiagnosis, with AD (30%), and psychiatric disorder (40%): depression (20%) and mania (20%), as main diagnostic categories. Similarly, in a previous Brazilian study, the most frequent misdiagnosis among FTD patients was psychiatric disorder followed by AD (Bahia, 2007). In general, the present study corroborated the finding of frequent misdiagnosis among FTD patients, especially confounding with AD and psychiatric diseases, observed in previous reports.^3,9,10,12,13,15^ The misdiagnosis with AD may be related to the difficulty encountered by clinicians and general practitioners in differentiating these dementias during the initial manifestation. Evidence on AD diagnostic criteria (NINCDS-ADRDA) has shown good sensitivity but poor specificity, contributing to diagnosis of other dementias such as FTD.^16^ The diagnostic value of the FTD consensus diagnostic criteria of Neary et al. (1998)^6^ showed high specificity and low sensitivity.^13^ This finding may also contribute to the difficulty in correctly assigning an FTD diagnosis and in including the AD diagnostic criteria.

In our study, patients with FTD had average delays of 4.4 years before receiving specialized care, which is similar to the average found by another Brazilian study (4.1 years).^17^ The delay for the expert evaluation was not only representative of this Southern referral center in Brazil, but has been reported for pre-senile dementias in general.^4,17^ This delay can jeopardize proper treatment and management of these patients and their families.

WND patients also had long duration of symptoms (4.3 years) where delay in accurate diagnosis was probably because most presented memory complaints (90%), but had no other feature fulfilling the criteria for dementia. The specialist evaluation found depression in 70% of these patients. Depressive patients often have memory deficits in the absence of other cognitive impairments.^18-20^ However, this evidence seems to be little disseminated among physicians since most tend to attribute memory complaints exclusively to AD. Therefore, these patients have received incorrect diagnosis, delaying proper treatment.

In the FTD group, the most consistent reason for referral was memory and behavior, followed by memory plus language problems. It seems that when these patients were not evaluated at specialized clinics complaints tended to be attributed to the diagnosis of AD and psychiatric disorders. A careful analysis of complaints and first symptoms could help reach proper diagnosis. The initial symptoms are extremely important for the differential diagnosis between different types of dementia and are important to fulfill diagnostic criteria.^6-8,13,14^

The cognitive symptoms reported in the specialist evaluation showed higher variability in the FTD group than in the other groups. The most frequent symptoms in FTD were language followed by memory problems. However, the FTD group was composed of more than one variant, allowing different cognitive manifestations. The core feature of the cognitive domain in PPAs is language, which is impaired early in the course of the disease. In initial phases, memory impairment is usually phonological and semantic, sparing episodic and visual memory as well as visuoperceptual abilities.^7,21^ At onset of disease, bvFTD patients can present relatively preserved performance on formal neuropsychological tests despite the presence of significant changes in personality and behavior.^8,22^ Impairment of executive function and a relative sparing of memory and visuospatial function can also be observed.^8,22^ Thus, cognitive symptoms found in our group of FTD patients were present according to the variants that comprised the group, such as bvFTD and PPAs, and according to the longer duration of the disease (i.e., more severe stages according to the CDR scale).

FTD patients showed worse outcomes after the 12-month period of follow-up (with institutionalization/worsening or death) than the other groups. In Brazil, another study had associated institutionalization with an unfavorable clinical course.^23^ Higher rates of behavioral and cognitive impairment, and higher degree of dependence in dementia, were also associated with higher rates of institutionalization.^23-25^ The worse outcomes observed in this study were correlated with delay in receiving proper diagnosis, which caused longer inadequate treatment, disease worsening, management difficulty of patients by family members, and institutionalization.

Additionally, we observed that most FTD patients received non evidence-based treatment, probably related to misdiagnosis (especially AD).

Limitations of the study were the small sample size of the groups studied and the use of the 1998 FTD diagnostic criteria. The small sample size was a consequence of the lower rates of FTD patients evaluated in dementia centers;^26^ consequently the present results should be interpreted cautiously. The 1998 FTD diagnostic criteria were applied since the current criteria were published in August 2011, after the present study period.

The results of this study pointed to the existence of difficulty by physicians (especially clinicians and general practitioners) in recognizing the main features of FTD and psychiatric disorders with memory impairment. Consequently, these professionals also delayed early referral to specialized centers and administering of appropriate treatment. We also observed that clinicians better recognized and dealt with AD. However, physicians tended to generalize memory complaints toward a single diagnosis, identifying almost all these patients as AD or leaving them undiagnosed. These findings suggest that patients with FTD evolved to worse outcomes than the other patients studied. Thus, diagnostic criteria and differential aspects of the diseases that cause cognitive impairment and dementia should be more widely disseminated.
